# Phage therapy in the Covid-19 era: Advantages over antibiotics

**DOI:** 10.1016/j.crmicr.2022.100115

**Published:** 2022-02-16

**Authors:** Atif Khan, T. Subba Rao, Hiren M. Joshi

**Affiliations:** a Water & Steam Chemistry Division, BARC Facilities, Kalpakkam, Tamil Nadu, India; b Homi Bhabha National Institute, Mumbai, Maharashtra, India

**Keywords:** SARS-CoV-2, Antibiotics, Multidrug resistance, Immunomodulation, Bacteriophages, Cytokines

## Abstract

Today, the entire world is battling to contain the spread of COVID-19. Massive efforts are being made to find a therapeutic solution in the shortest possible time. However, the research community is becoming increasingly concerned about taking a shortsighted strategy without contemplating the long-term consequences. For example, It has been reported that only 8.4% of total COVID-19 patients develop a secondary bacterial infection. In comparison, 74.6% of them are administered with antibiotics as prophylactic treatment. We contend that overuse of broad-spectrum antibiotics increases the likelihood of AMR development and negatively affects the patient's recovery due to the prevalence of the "gut-lung axis.". Consequently, the use of antibiotics to treat COVID-19 patients must be rationalized, or an alternative treatment must be sought that does not risk contributing to AMR development and positively impacts the treatment outcomes. Phage therapy, a century-old concept, is one of the most promising approaches that can be adapted to serve this purpose. This review emphasizes the negative impact of excessive antibiotic use in COVID-19 treatment and provides an overview of how phage therapy can be used as an alternative treatment option. We have argued that targeted killing (narrow spectrum) and anti-inflammatory (which can target the primary cause of mortality in COVID-19) properties of phages can be an effective alternative to antibiotics.

## Highlights

• Excessive use of antibiotics during the COVID-19 pandemic may accelerate the emergence of AMR.
• Antibiotic-mediated dysbiosis significantly impacts immune-homoeostasis and thereby negatively impacting the recovery from COVID-19.
• Antibiotic-induced dysbiosis will also negatively impacts the pulmonary functioning of the COVID-19 patient via the Gut-Lung Axis.
• Bacteriophages or “phage therapy” can be an ideal alternative for antibiotics having desired specificity and availability.
• Phage therapy can also act as an anti-inflammatory agent to avoid cytokine storm in COVID19.

## Graphical abstract

**Figure:**
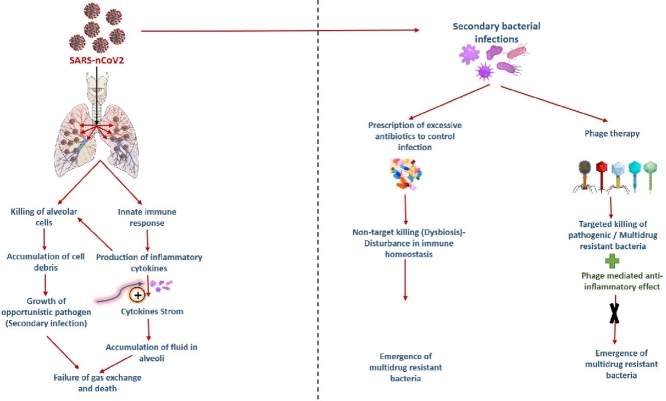

## 1 Introduction

Over the last 2000 years, the human race has faced a number of epidemics/pandemics that have killed millions of people and resulted in significant economic losses. The frequency of epidemics has increased significantly over the last century. Recent examples include Asian flu, AIDS, Swine flu, Ebola, Zika virus infection, and current SARS-CoV-2 or COVID-19 infections. Globalization and international travel have accelerated the spread of these infections, with some of them rapidly reaching pandemic proportions. Unfortunately, most of these epidemics or pandemics are of viral origin, and new antiviral therapeutics are still being developed (Richman and Nathanson, 2016). Despite the recent pandemic of Severe Acute Respiratory Syndrome (SARS), we do not have a universal therapeutic agent or any promising drug candidate against these severe acute respiratory syndrome (except antibody cocktails and recently developed drugs like Paxlovid and Molnupiravir) that can be quickly deployed to treat prevailing SARS-CoV-2 infection (Dolgin, 2021).

The current pandemic was caused by SARS-CoV-2, which first appeared in the Chinese province of Hubei in November 2019 and spread throughout the world over the next few months (Zhang et al., 2020). The SARS-CoV-2 virus is highly contagious and spreads through human-to-human contact. The respiratory system is the primary site of infection for the virus. However, growing evidence suggests that multiple organs, including the heart, brain, kidney, and digestive system/organ, are involved (Gavriatopoulou et al., 2020; Peiris et al., 2021). The adaptive immune system plays a critical role in combating viral attacks on our bodies by producing neutralizing antibodies against viral antigens. However, the adaptive immune system's response weakens with age or any other physiological infirmity, causing a delay in the production of antiviral antibodies. (Weyand and Goronzy, 2016). In the case of SARS-CoV-2 infection, any delay in activation of adaptive immune response results in an aggressive inflammatory response by the innate immune system (inflammatory cytokine storm). This inflammatory response has the potential to cause severe lung damage and subsequent secondary bacterial infection. As a result, prescribing antibiotics in addition to standard antiviral therapy as a prophylactic measure to prevent secondary bacterial infections is being adopted around the world. (Beović et al., 2020; Grau et al., 2021; Yacouba et al., 2021). However, a meta-analysis found that only 8.4% of COVID-19 patients were diagnosed with a secondary bacterial infection. On the contrary, 74.6% of patients are given antibiotics as a preventative measure to avoid secondary infection (Langford et al., 2021). This scenario suggests that antibiotics are being used inappropriately and possibly excessively in COVID treatment, which may result in a significant increase in antibiotic-resistant organisms in a short period of time (Founou et al., 2021; Livermore, 2021). Antibiotic resistance is already predicted to become a pandemic by 2050, causing 10 million deaths per year worldwide. Antibiotic overuse in the COVID-19 pandemic may hasten this predicted scenario (Tagliabue and Rappuoli, 2018). In addition, antibiotics have several indirect effects on our immune system. For example, antibiotics mediated extensive killing of gut microbiota (i.e. dysbiosis) is known to have a negative impact on immune homeostasis (Becattini et al., 2016; Sun et al., 2019). Therefore, it is highly desirable to have calibrated approach for antibiotics usage or find alternative therapeutics for infection management. The new therapeutic(s) should be free from the associated danger of resistance development and have properties that synergistically or positively impact our immune system.

Bacteriophages are naturally occurring in the environment and are highly specific in their ability to kill host bacteria (Batinovic et al., 2019). Frederick Twort and Felix d'Herelle independently discovered them in 1915 and 1917. During the 1920s and 1930s, bacteriophages were successfully used to treat various bacterial infections and termed as "Phage therapy". However, during WWII, the mass production of antibiotics and rapid expansion of their use resulted in a significant loss of interest in phage therapy (Fernández et al., 2019). Nonetheless, the recent emergence of AMR has brought phage therapy back into the spotlight. Many recent reports have highlighted the successful use of bacteriophages in clinical and non-clinical settings to eliminate antibiotic-resistant bacterial strains (Chan et al., 2021; Chang et al., 2018; Ke et al., 2019; Taati Moghadam et al., 2020; Wittebole et al., 2014). Historical evidence, combined with newly published data, provides a solid foundation for implementing phage therapy to control secondary bacterial infection in SARS-CoV-2 patients without the danger of causing AMR (Wojewodzic, 2020). This review is an honest attempt to highlight the negative impact of overuse of antibiotics in COVID-19 treatment and how phage therapy can be a promising way to treat secondary bacterial infection in COVID-19 patients without significantly accelerating AMR development.

## 2 Antibiotic treatment for secondary bacterial infection in COVID-19: A inflexion point of antibiotics resistance development

Though the pathogenesis of SARS-CoV-2 is still being studied, it can be divided into two distinct phases. 1) viral replication phase and 2) uncontrolled inflammatory immune responses (Trougakos et al., 2021). The inflammatory phase is extremely critical for the patient and may lead to mortality if not treated in advance. Emerging evidence suggests that COVID-19 patients have a miscommunication between innate and adaptive immune systems, resulting in immune hyperactivity (Langford et al., 2020). During the second stage of COVID-19 infection, heightened inflammatory response causes extensive damage to alveolar cells. The damaged alveolar cell provides a nutrient-rich environment for opportunistic pathogens to thrive. The proliferation of opportunistic pathogens starts a new cycle of the inflammatory response (cytokine storm), which causes excessive fluid accumulation in the lungs and, as a result, complete loss of the gas exchange process (i.e. pulmonary destruction) (Ragab et al., 2020). Consequently, majority of clinicians around the world are prescribing antibiotics in combination with other supportive treatments to COVID-19 patients to limit the risk of secondary bacterial infection and inflammatory immune response. However, a comparison of individuals who develop secondary infections and those administered with antibiotics shows that antibiotics are being used excessively. For example, 216 million excess doses of antibiotics were prescribed in India during June-September 2020 (Sulis et al., 2021). Similarly, A scoping review by Cong et al. indicated 51.4% unjustified antibiotics prescription to the COVID-19 patients in the first phase of the COVID-19 pandemic (Cong et al., 2021). Although there is a higher risk of secondary bacterial infection in patients with severe covid- 19, it should only be used after a thorough evaluation. Before deciding on an antibiotic treatment plan, a clinician must recommend few tests like blood culture with antibiotic sensitivity test, pneumococcal urinary antigens, procalcitonin test (PCT) etc. (Jolobe, 2021). However, it has been reported that many patients were given antibiotics despite having negative blood culture results (Bhatt et al., 2021). This overuse of antibiotics may result in an excessive discharge into water bodies, contributing to the spread of AMR (Rizzo et al., 2013). Reports of elevated concentrations of antibiotics in wastewater treatment plants and a significant increase in antibiotic resistance in water bodies have already started appearing in publications (Comber et al., 2020; Kumar et al., 2021). At this juncture, we must remember the cautionary words from the discoverer of antibiotics, Alexander Fleming while accepting the Nobel prize "*I would like to sound one note of warning. The time may come when penicillin can be bought by anyone in the shops and ignorant person may underdose himself to non-lethal quantities of antibiotics making the microbes resistant"* and needs to take all necessary steps to avoid the catastrophic impact of a sudden rise in AMR (Rosenblatt-Farrell, 2009).

### 2.1 Antibiotics as immunomodulators

In addition to the direct impact of antibiotic usage during COVID-19 on AMR development, there are many indirect effects one needs to keep in mind before prescribing antibiotics. For example, most antibiotics used as prophylactic treatment for secondary bacterial infection are broad-spectrum antibiotics that can eliminate bacteria other than the targeted ones. This non-specific, broad-range killing also affects gut microbes and can result in "dysbiosis", a condition characterized by compositional and functional changes in the gut microbiome. (Levy et al., 2017). It is well established that the gut microbiota plays a vital role in immune homeostasis; thus, any significant change in the composition of gut microbes may result in a shift in immune response (Belkaid and Hand, 2014). Gut dysbiosis, for example, has been linked to autoimmune and inflammatory disorders such as Rheumatoid arthritis, inflammatory bowel disease, type 1 diabetes, etc. (Dhar and Mohanty, 2020; Li et al., 2018). Reports have shown a significant shift in gut microbiota towards opportunistic pathogens in COVID-19 patients with extensive gut dysbiosis (Chen and Vitetta, 2021; Yamamoto et al., 2021a; Zuo et al., 2020).

Though there is no direct evidence linking gut microbiota to COVID-19 progression, the available evidence on the prevalence of "Gut-Lung Axis" suggests that it might have a substantial impact (Bradley et al., 2019; Keely et al., 2012; Steed et al., 2017; Yamamoto et al., 2021b). "Gut-Lung Axis" refers to the cross-talk between gut microbiota and lungs and is shown to impact the progression of various respiratory infections. According to Steed et al., microbiota-associated metabolites protect against influenza infection via type 1 interferon (Steed et al., 2017). They have shown that desaminotyrosine produced by gut microbes protects mice from influenza. This protection was diminished when the mice were treated with antibiotics due to antibiotic-mediated dysbiosis. ACE2 receptors are the primary cell entry receptor by which SARS-Cov-2 enters the cell, and therefore, any variation in expression of ACE2 will directly impact the progression of COVID-19. Koester et al. have shown that gut microbes directly influence the expression of ACE2 receptors. They have used expression profiling of ACE2 receptors in the presence or absence of different microbiota to establish that an increase in gut microbial diversity decreases the expression of ACE2 receptors while antibiotics mediated dysbiosis increases the expression of ACE2 receptors (Koester et al., 2021). On a similar note, Hashimoto *et al*. have established a linkage between ACE2, dietary amino acids and gut microbiota (Hashimoto et al., 2012). They discovered that ACE2 directly regulates the luminal microbiome by regulating the secretion of antimicrobial peptides. Similarly, ACE2 was discovered to be in charge of regulating the renin-angiotensin system (RAS), which has been linked to lung damage, cardiovascular disease, and SARS infection. Dang and Marsland's excellent review summarises all the reported evidence indicating the prevalence of the "Gut-Lung Axis" (Dang and Marsland, 2019). However, the molecular mechanism underlying the relationship between luminal microbiota and ACE2 expression is not well understood and warrants further investigation. Given the direct and indirect effects of antibiotics on the immune system and the inherent risk of accelerated AMR development, recalibrating the antibiotic treatment protocol for COVID-19 patients is critical. Similarly, one should seek out an effective alternative therapy to replace or supplement antibiotics.

## 3 Phage therapy as a promising candidate for the treatment of secondary infections in COVID-19

Bacteriophages are viruses that specifically kill their bacterial hosts. They are also known as phages or bacterial viruses. For adsorption and infection, they recognize specific cell surface receptors such as peptidoglycan, lipopolysaccharide, and transport proteins on their host cell surface (Bertozzi Silva et al., 2016; Rakhuba et al., 2010). They are omnipresent and found across different habitats like soil, river, sea, desert etc. They are also associated with various excretions like faces, phlegm and body fluids. Bacteriophages play an important role in maintaining community dynamics and bacterial diversity via the "kill the winner" (KtW) phenomenon (Maslov and Sneppen, 2017). According to this theory, bacteriophages contribute to population diversity by selectively eliminating dominant bacterial species. As per one estimation, more than 10^23^ phages are infecting their hosts on the planet at any given time (Suttle, 2007).

The bacteria-killing property of bacteriophages can be used to selectively eliminate pathogenic bacteria. They can be an excellent antibiotic replacement because of their host specificity and ease of isolation from the environment. Bacteriophages are known to outperform antibiotics in the treatment of bacterial infections caused by bacterial biofilms. Bacteria in biofilms are naturally resistant to antibiotics due to their unique penetration barriers (i.e. extracellular polymeric substances do not allow antibiotics to reach the cells). Furthermore, physiological heterogeneity, reduced growth rate, hypoxia, stringent response, quorum sensing, and genetic factors like horizontal gene transfer and mutation play an important role in conferring antibiotic resistance to biofilms (Hall and Mah, 2017). Unfortunately, biofilms are responsible for two-thirds of human infections, making antibiotic treatment extremely difficult (Harper et al., 2014). In contrast to antibiotics, bacteriophages effectively eradicate biofilms due to the presence of EPS-degrading enzymes like endolysins and depolymerase in their tails (Ferriol-González and Domingo-Calap, 2020). This active penetration boosts bacteriophage effectiveness in biofilm-related infections. Furthermore, like antibiotics, bacteriophages provide the flexibility to treat bacterial infections with a single phage or a mixture of multiple phages (i.e. phage cocktail) to eradicate polymicrobial infection.

### 3.1 Resistance development: Phage therapy V/s antibiotic therapy

Bacteria have evolved numerous mechanisms for acquiring antibiotic resistance. A few of them are determined by genetic changes such as mutation and horizontal gene transfer. While some of them are controlled by physiological factors such as enzymatic modification or destruction of the antibiotic molecule, change in cell permeability, efflux pump, etc. (Munita and Arias, 2016). Antibiotics have broad-spectrum activity and are thus used to treat a wide variety of bacterial pathogens. A few of them may develop resistance mechanisms and pass them on to others through horizontal gene transfer (resistant donor). Due to Phage's host specificity, distantly related bacteria (non-host) are unlikely to have ever been exposed to the Phage of interest and thus are unlikely to contribute resistance genes to non-host via horizontal gene transfer (Cohan et al., 2020a). Likewise, CRISPR-based phage immunity is unlikely to protect other taxa and thus does not contribute to resistance spread via HGT (Cohan et al., 2020b). Therefore, the likelihood of phage resistance development and spread is significantly lower than that of antibiotic resistance development. Furthermore, it has been reported that when antibiotics and bacteriophages are used together, they have a synergistic effect because targeted bacterial strains find it challenging to overcome two different stresses simultaneously (Gu Liu et al., 2020). Chan *et al*. have shown an interesting approach where they have used bacteriophages to increase the antibiotic sensitivity of multidrug-resistant *Pseudomonas aeruginosa*. The approach involves careful selection of bacteriophage and antibiotic in a way that their respective resistance development remains incompatible with each other. In other words, resistance development against bacteriophage will make the bacterium susceptible to antibiotics or vice versa. They have shown that *P.aeruginosa* evolves phage resistance by modifying outer membrane porin M (OprM), making them more susceptible to antibiotics (Chan et al., 2016). Additionally, the prevalence of pleiotropy among cell targets responsible for antibiotic resistance and phage attachment provides an additional safety mechanism that limits the development of resistance when antibiotics and bacteriophages are used together (Burmeister et al., 2020). As listed in Table 1, there are multifold advantages of bacteriophages over antibiotics for infection control.

**Table 1:** Comparative advantages of bacteriophages over antibiotics for infection control.

Criteria	Antibiotics	Bacteriophages
Specificity to bacteria	Non-specific, Non-target organisms, including normal flora of the body, gets killed.	Highly specific for its bacterial host and does not disturb beneficial bacteria.
Resistance	Broad-spectrum antibiotics are repeatedly used to treat various infections and therefore, more chances of resistance development.	Phage specificity limits the use of specific phage and therefore, limited chances of resistance development
Mode of action	Inhibitory action over DNA, RNA, Protein, or cell wall synthesis	Cell lysis
Effectivity on bacterial biofilms	Less effective due to penetration barrier and biofilm-associated antibiotics resistance	Most phages have EPS degrading enzymes like depolymerase, which help them to penetrate and kill the host
Dose	Systemic dose (i.e., equally distributed in the entire body).	The higher number near the site of infection (targeted therapy)
Effect on the immune system	Direct effect by many immunomodulatory antibiotics and indirect effect via dysbiosis	Highly purified phage preparations have a negligible immune response. Few phages act as an immunomodulator
Environmental impact	An environmental release may lead to waterbody contamination and the development of antibiotic resistance	The shorter life (outside host) and non-availability of a host lead to rapid elimination from the environment.

**Fig. 1:**
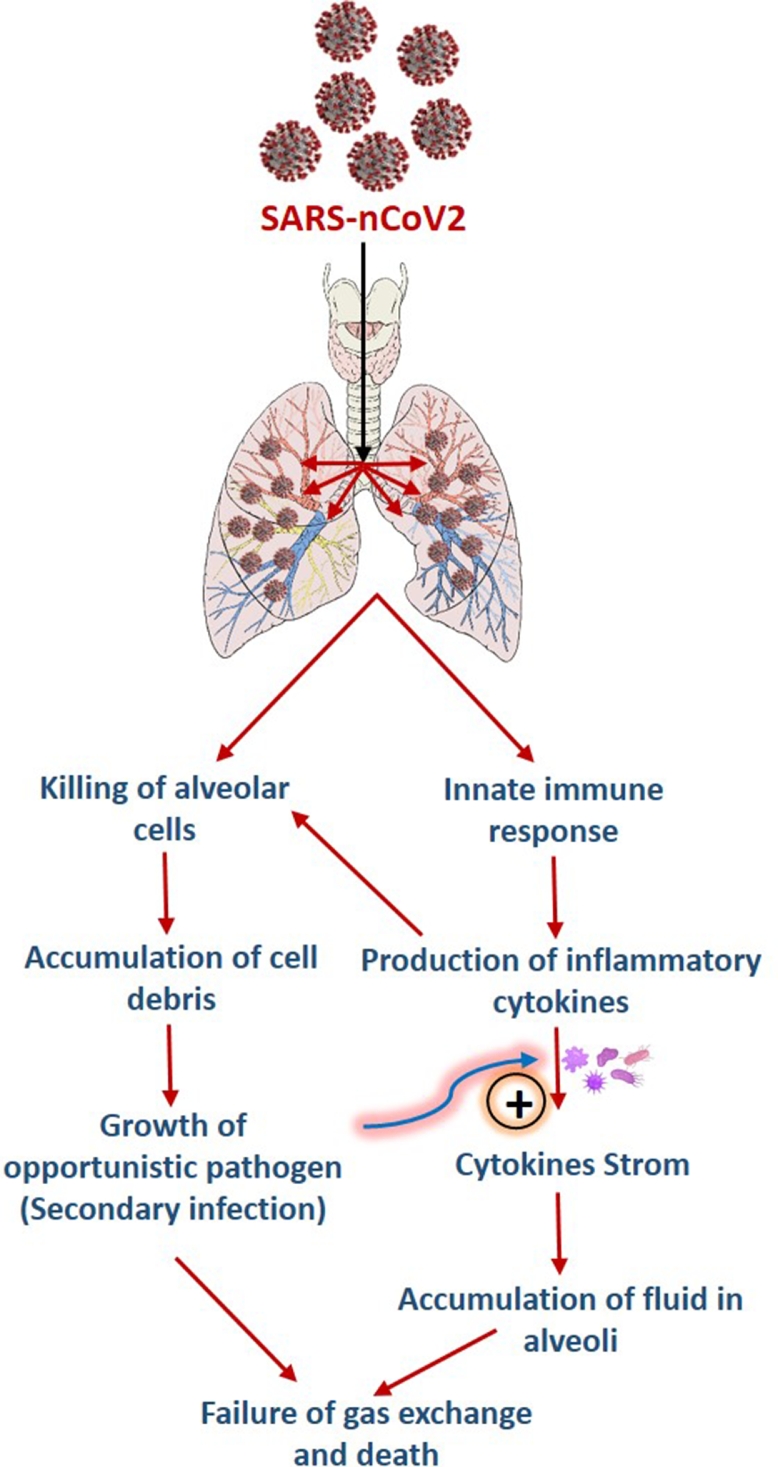
A schematic illustration showing the infection of SARS-CoV-2 in alveolar cells, causing secondary bacterial infections.

**Fig. 2:**
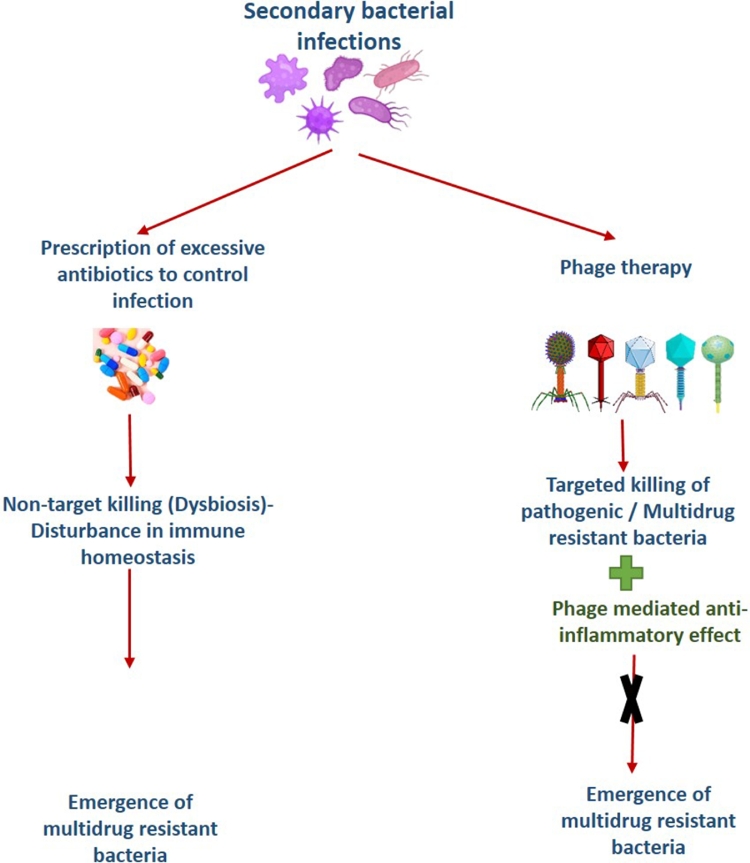
Comparative advantages of Phage therapy over antibiotics for treating secondary bacterial infection.

In 1919, the first documented use of bacteriophage to treat bacterial infections was reported, and then after, there have been numerous reports on the successful use of bacteriophages in clinical and pre-clinical settings for infection control (Sulakvelidze et al., 2001; Vandamme and Mortelmans, 2019). Recently, Wu et al. have successfully demonstrated the efficacy of phage therapy against secondary infection of *Acinetobacter baumannii* in COVID-19 patients (Wu et al., 2021). This observation firmly establishes the applicability of phage therapy for treating secondary bacterial infection in COVID-19 patients. Therefore, accelerated effort should be made to bring phage therapy as a mainstream treatment alternative to treat secondary infection in COVID-19 patients Fig. 1, Fig. 2. depicts a schematic of how bacteriophages can be used to control secondary bacterial infection in COVID-19 patients without causing antibiotic-resistant organisms to emerge.

### 3.2 Advantage of phage therapy as an immunomodulator in COVID-19

Inflammation is a hallmark of severe SARS-CoV-2 infection, manifested by increased inflammatory biomarkers such as C Reactive Proteins (CRP), Lactic Dehydrogenase (LDH), ferritin, d-dimer, IL-6, and others, resulting in a cytokine storm (García, 2020). Interestingly, the most promising aspect of phage therapy is its anti-inflammatory properties. Miedzybrodzki et al. discovered that phage therapy with T4 phages significantly reduced inflammatory cytokines and C reactive proteins (Miedzybrodzki et al., 2009). Cafora et al. found that the proteinaceous component of a *Pseudomonas aeruginosa* specific phage has anti-inflammatory activity in a zebrafish cystic fibrosis model. They have shown that Toll-like receptor (TLR) pathway is involved in this anti-inflammatory activity. (Cafora et al., 2020). Pabary et al. made a similar observation in a murine model where they have shown a significant reduction in inflammatory cytokines in bronchoalveolar lavage fluid (BALF) (Pabary et al., 2016).

There are two possible mechanisms for Phage's anti-inflammatory action: 1) indirect effect by reducing bacterial load by directly killing the targeted pathogen or assisting their clearance from the body and 2) direct effect by interacting with the host immune system. In addition to direct killing (lysis) of their respective host, bacteriophages also helps in clearing them out from the body. For example, Phage increases the phagocytosis of its bacterial host by opsonization. This assisted phagocytosis helps in the early clearance of pathogenic bacteria (Van Belleghem et al., 2018). Similarly, bacteriophage adherence to mucus (BAM) provides a unique non-host derived innate immunity where phages embedded in mucus provides natural defense against their pathogenic host (Barr et al., 2013). The rapid clearance of pathogens from the host body via direct lysis, assisted phagocytosis, and BAM reduces the bacterial load and thus inflammation (Hill et al., 2000; Huffnagle et al., 2017). Furthermore, evidence of direct interaction between the immune system and bacteriophages is accumulating. For example, evidence suggests that Phage plays a critical role in reducing ROS production by phagocytic cells, altering the expression of cell surface markers, inhibiting immune cell migration, and modulating cytokine secretion (Górski et al., 2021). Various in vitro and in vivo studies suggest that Phage's anti-inflammatory activity is primarily governed by interference with the expression of the nuclear transcription factor NF-kappa B (Górski et al., 2016). It has also been demonstrated that the tail protein of T4 phages reduces the immunogenicity of LPS when it binds to LPS of bacterial cell wall (Górski et al., 2006; Miernikiewicz et al., 2016; Przybylski et al., 2015). Gorski et al. provided an excellent review that details the close interaction between bacteriophages and the human immune system (Górski et al., 2019) Fig. 3. summarises the various interactions of bacteriophages with the immune system as well as the possible mechanisms by which they act as immune modulators. Based on the preceding discussion, it appears that phage therapy can serve two functions in the treatment of Covid-19: a) control of secondary bacterial infection and b) anti-inflammatory agent to control cytokine storm. These two distinct advantages of phage therapy strongly support its use in the treatment of COVID-19.

**Fig. 3:**
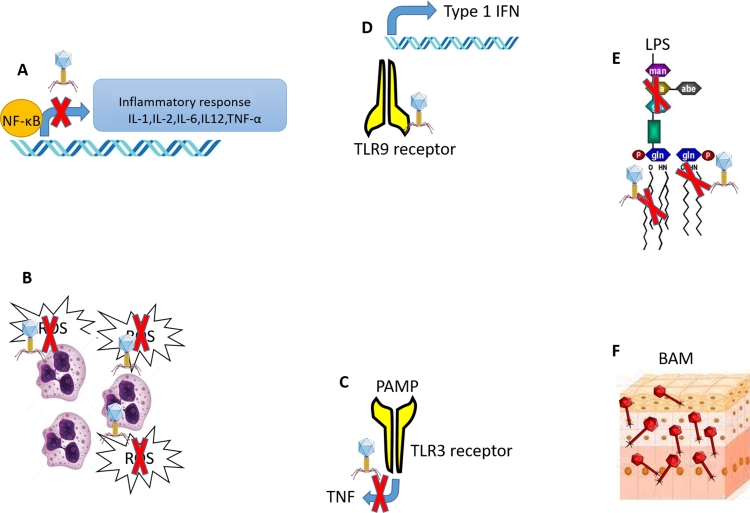
Interaction of bacteriophages with our immune system . A) Inhibition of NF-κB mediated expression of pro-inflammatory cytokines, B) Inhibition of ROS generation, C) Inhibition of expression of TNF via Toll-like receptors, D) Induction of Type 1 interferon via Toll-like receptor, E) Direct binding to LPS and F) non–host-derived immunity via Bacteriophage Adherence to Mucus.

### 3.3 Limitations of phage therapy

Though phage therapy is one of the most promising technologies for combating antibiotic-resistant infections, many obstacles must be overcome before being used as a routine therapeutic agent. For example, there is a lack of reliable data on clinical trials and regulatory guidelines for phage-based therapeutics (Liu et al., 2021). Secondly, one can observe significant variation in efficacy of phage therapy within the test group. This disparity in efficacy within the test group is primarily due to our limited understanding of in vivo pharmacokinetics and pharmacodynamics of bacteriophages (Nilsson, 2019). Furthermore, the immunogenicity of a given phage must be thoroughly evaluated prior to application in order to avoid immunogenic clearance of phages (Loc-Carrillo and Abedon, 2011). The Phage's narrow specificity limits its applicability to a single strain of an organism. As a result, multiple phages must be screened to identify that strain-specific Phage. However, there are multiple phages specific to strain of interest and, therefore, can be easily isolated from the environment. Other critical aspects that need to be considered before selecting a phage for treatment include phage resistance and the lysogenic life cycle of a given phage (Berryhill et al., 2021). Even though phage therapy has few limitations, given the rapid rise in antibiotic resistance and the limited supply of new antibiotics, we must embrace this technology to combat bacterial infection. Data from various ongoing clinical trials and active research in various laboratories will assist us in overcoming those limitations. Additionally, we urgently need to change our perception of this century-old technology, and many more research laboratories must contribute to its rapid deployment as anti-infection therapy.

## 4 Conclusion

The COVID-19 pandemic has presented us with numerous challenges, and the entire world is striving to overcome this situation at the earliest. The rapid development of vaccines in record time has given us hope that we will be able to overcome this pandemic soon. However, we must also consider the long-term consequences of this pandemic and plan accordingly. Excessive use of antibiotics during this pandemic is one of the major concerns shown by the research community. Today, clinicians across the world are prescribing antibiotics to prevent secondary bacterial infection in Covid-19 patients. However, data suggest that most of the time, this prophylactic use of antibiotics is not justified. On the contrary, this excessive usage has significantly increased the possibilities of the accelerated emergence of antibiotic-resistant organisms. As a result, we must recalibrate our approach towards treating the patient with antibiotics and increase our efforts to develop an alternative infection control strategy. Phage therapy having numerous success stories can be an ideal alternative.The rapid expansion of antibiotic production during World War II has completely diverted our attention from this century-old technology. Now the time has come to bring it back as an alternative to antibiotics. Bacteriophages with high specificity (targeted killing) and anti-inflammatory properties will be of great value to treat a disease like Covid-19.

## Credit author statement

Atif Khan: Writing - Original Draft

Hiren Joshi: Conceptualization, Writing - Review & Editing

T. Subba Rao: Supervision, Project administration

## Declaration of Competing Interests

The authors declare that they have no known competing financial interests or personal relationships that could have appeared to influence the work reported in this paper.

## Availability of data and material

Not applicable

## Code availability

Not applicable

## Declaration of Competing Interest

The authors declare that they have no known competing financial interests or personal relationships that could have appeared to influence the work reported in this paper.
